# The fetal lineage is susceptible to Zika virus infection within days of fertilization

**DOI:** 10.1242/dev.200501

**Published:** 2022-07-28

**Authors:** Jennifer L. Watts, Amy Ralston

**Affiliations:** 1 Molecular, Cellular and Integrative Physiology Graduate Program, Michigan State University, East Lansing, MI 48824, USA; 2 Reproductive and Developmental Biology Training Program, Michigan State University, East Lansing, MI 48824, USA; 3 Department of Biochemistry and Molecular Biology, Michigan State University, East Lansing, MI 48824, USA

**Keywords:** Zika virus (ZIKV), Birth defects, Miscarriage, Developmental biology, Cell fate, Epidemic

## Abstract

Adults contracting Zika virus (ZIKV) typically exhibit mild symptoms, yet ZIKV infection of pregnant individuals can cause miscarriage or birth defects in their offspring. Many studies have focused on maternal-to-fetal ZIKV transmission via blood and placenta. Notably, however, ZIKV is also transmitted sexually, raising the possibility that ZIKV could infect the embryo shortly after fertilization, long before the placenta is established. Here, we evaluate the consequences of ZIKV infection in mouse embryos during the first few days of embryogenesis. We show that divergent strains of ZIKV can infect the fetal lineage and can cause developmental arrest, raising concern for the developmental consequences of sexual ZIKV transmission.

This article has an associated ‘The people behind the papers’ interview.

## Abstract (precis)

**Summary:** Mouse preimplantation embryos are vulnerable to Zika virus-induced lethality even in the presence of the zona pellucida, highlighting a potential risk of sexually transmitted infection in early pregnancy.

## INTRODUCTION

The Zika virus (ZIKV) is a zoonotic member of the *Flaviviridae* family that usually causes relatively mild symptoms in adults including fever, rash and joint pain (Edupuganti et al., 2017). However, in some pregnancies, vertical transmission of ZIKV from mother to fetus results in birth defects or miscarriage, whereas other pregnancies are unaffected (Brasil et al., 2016; del Campo et al., 2017; Honein et al., 2017; Pacheco et al., 2020; Soares de Souza et al., 2016; van der Eijk et al., 2016). The reasons for the widely varying pregnancy outcomes are unclear, but could include human genetic variation, prior exposure to flaviviruses or, most relevant to developmental biologists, the timing of ZIKV infection during pregnancy (Cao et al., 2017; Platt et al., 2018; Zorrilla et al., 2017).

Less commonly discussed is the role that the route of infection bears on pregnancy outcomes. Prior studies have primarily focused on the descending route of vertical transmission, from mother to fetus via the placenta (Zanluca et al., 2018). Fewer studies have focused on the ascending route of infection, wherein virus is transmitted to developing offspring within the maternal reproductive tract (Carroll et al., 2017; Counotte et al., 2018; Stassen et al., 2018; Yockey et al., 2016). As ZIKV is sexually transmitted (Brooks et al., 2016; D'Ortenzio et al., 2016; Grischott et al., 2016), ZIKV infection of embryos could occur shortly after fertilization. Nevertheless, the effects of ZIKV on early embryonic development are still understudied.

We hypothesize that, prior to implantation, the embryo is vulnerable to viral infection, because neither the placenta nor the adaptive immune system has yet developed. For several days following fertilization, preimplantation embryos develop as free-floating entities within the female reproductive tract. During these stages, crucial developmental events occur, including establishment of the fetal lineage, as well as extra-embryonic lineages, such as the yolk sac and placenta (Chazaud and Yamanaka, 2016). During preimplantation development, the embryo is surrounded by a thick glycoprotein coat called the zona pellucida (ZP). However, it is currently unknown whether the ZP protects embryos from viral infection throughout preimplantation stages.

How ZIKV infection affects preimplantation development is enigmatic; to date only two studies have explored this topic. One study evaluated the effects of a Puerto Rican strain of ZIKV on preimplantation rhesus monkey embryos (Block et al., 2020), and another evaluated the effects of a Ugandan strain of ZIKV on mouse embryos (Tan et al., 2019). Both studies concluded that ZIKV exposure can be lethal to blastocysts lacking the ZP. However, differences in experimental design, including embryo species, presence of the ZP, viral strain, and analysis endpoints, make comparison of these studies challenging. Here, we present a systematic evaluation of lineage-specific markers of mouse preimplantation embryos exposed to ZIKV in the presence and absence of the ZP. We report that the ZP can protect preimplantation embryos from ZIKV-induced lethality at some developmental stages, whereas other embryonic stages are vulnerable to ZIKV-induced lethality even when the ZP is intact.

## RESULTS

### ZIKV can infect all blastocyst lineages, including the fetal lineage

**Fig. 1.**
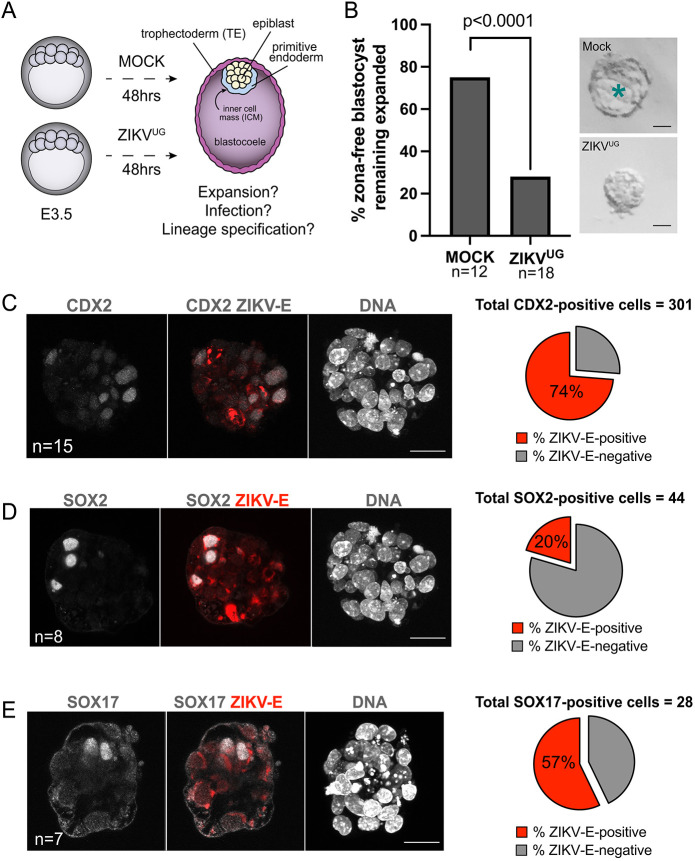
**ZIKV^UG^ causes defects in blastocyst development.** (A) Schematic of the experimental design. Embryos were harvested at E3.5 (blastocyst stage), the ZP was removed, then embryos were transferred to embryo culture medium containing ZIKV^UG^ or mock medium and allowed to develop until endpoint analyses. (B) Proportion of blastocysts remaining expanded, with representative images. Asterisk indicates the blastocoel. Statistical test: χ^2^. (C) Maximum projection of all sections of *z*-stack confocal imaging of CDX2 and ZIKV-E immunofluorescence and nuclear stain for a representative ZIKV^UG^-infected blastocyst. Pie chart shows the proportion of ZIKV-E-positive/negative CDX2-positive cells across all embryos. (D) Maximum projection of all sections of *z*-stack confocal imaging of SOX2 and ZIKV-E immunofluorescence and nuclear stain for a representative ZIKV^UG^-infected blastocyst. Pie chart shows the proportion of ZIKV-E-positive/negative SOX2-positive cells across all embryos. (E) Maximum projection of all sections of *z*-stack confocal imaging of SOX17 and ZIKV-E immunofluorescence and nuclear stain for a representative ZIKV^UG^-infected blastocyst. Pie chart shows the proportion of ZIKV-E-positive/negative SOX17-positive cells across all embryos. *n*, number of embryos. Scale bars: 20 µm (B); 25 µm (C-E).

A prior study reported that some cells of wild-type mouse preimplantation embryos can be infected by exposing blastocysts [embryonic day (E) 3.5] to 6×10^4^ plaque-forming units (pfu)/ml of a Ugandan strain of ZIKV (ZIKV^UG^), when the ZP is removed (Tan et al., 2019). Consistent with this observation, we observed significantly (*P*<0.0001) compromised developmental progression in ZP-removed embryos exposed to ZIKV^UG^ at E3.5 (*n*=18), compared with mock-infected ZP-free embryos (*n*=12) (Fig. 1A,B). For all experiments, ZIKV was raised in Vero cells, and then the conditioned medium was diluted into embryo culture medium to achieve the indicated viral concentrations. For negative control experiments, Vero cell medium or Vero cell-conditioned medium was diluted into the embryo culture medium at the exact dilution as for the infected embryos, as has been done previously (Block et al., 2020; Scaturro et al., 2018).

Next, we evaluated the localization of the ZIKV viral envelope protein (ZIKV-E) within cells of the embryo, as an indicator of infection (Tan et al., 2019). Because the blastocyst contains three distinct cell types, we evaluated infection of each cell type individually. First, we focused on trophectoderm cells. The trophectoderm, which contains progenitors of the placenta, surrounds the blastocyst and is specifically labeled by the essential transcription factor CDX2 (Strumpf et al., 2005). We detected ZIKV-E within CDX2-positive cells of blastocysts after ZIKV^UG^ exposure (Fig. 1C), but not in mock-infected, control blastocysts (Fig. S1), consistent with prior observations (Tan et al., 2019).

We then evaluated ZIKV-E within the epiblast, which has not been previously examined in blastocysts. Epiblast cells are the pluripotent progenitors of the entire fetus and are located within the inner cell mass (ICM) of the blastocyst. At this stage, epiblast cells comprise about half of all ICM cells, and are intermixed with cells of the primitive endoderm (Chazaud et al., 2006), an extra-embryonic lineage that is crucial for axial patterning, germ cell specification, and development of cardiac, blood and intestinal cells (Belaoussoff et al., 1998; de Sousa Lopes et al., 2004; Kwon et al., 2008; Madabhushi and Lacy, 2011; Mao et al., 2010; Nowotschin et al., 2019; Thomas and Beddington, 1996). Epiblast cells can be discerned within the ICM on the basis of SOX2 expression (Wicklow et al., 2014). Remarkably, ZIKV^UG^ was detected within SOX2-positive cells of blastocysts exposed to ZIKV^UG^ (Fig. 1D).

Finally, we evaluated ZIKV-E within the primitive endoderm, which has not been previously examined. In the blastocyst, primitive endoderm cells can be identified on the basis of SOX17 expression (Plusa et al., 2008). ZIKV-E was detected in SOX17-positive primitive endoderm cells in blastocysts (Fig. 1E). Therefore, all three blastocyst lineages are infected by ZIKV^UG^.

### ZIKV^UG^ infection disrupts cell fate in the blastocyst

**Fig. 2.**
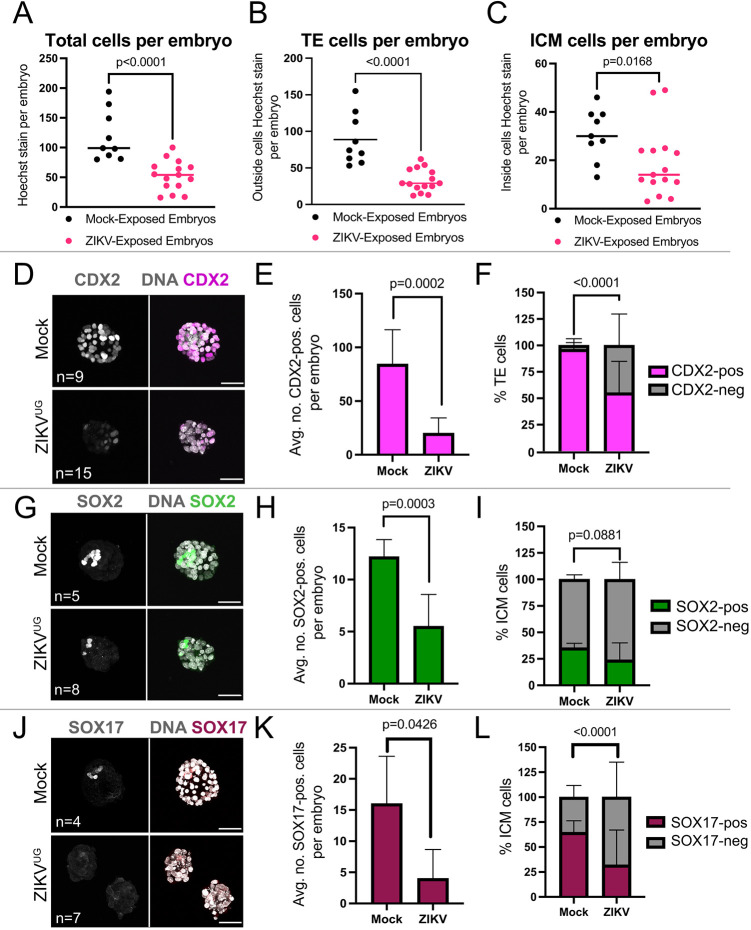
**ZIKV^UG^ disrupts cell fate specification in the blastocyst.** (A-C) Total number of cells (A), number of trophectoderm (TE) cells (B) and number of ICM cells (C) in each blastocyst (harvested at E3.5) after 48 h in mock and ZIKV-infected conditions. Statistical test: unpaired *t*-test. Horizontal line indicates the mean. (D) Maximum projection of all sections of *z*-stack confocal imaging of CDX2 immunofluorescence and nuclear stain for a representative mock-infected and a representative ZIKV^UG^-infected blastocyst. (E) Average number of CDX2-positive cells in all embryos (sample sizes provided in the image panels) for each condition. Statistical test: unpaired *t*-test. (F) Proportion of outside cells (TE cells) in which CDX2 was detected among blastocysts. Statistical test: χ^2^. (G) Maximum projection of all sections of *z*-stack confocal imaging of SOX2 immunofluorescence and nuclear stain for a representative mock-infected and a representative ZIKV^UG^-infected blastocyst. (H) Average number of SOX2-positive cells in all embryos (sample sizes provided in the image panels) for each condition. Statistical test: unpaired *t*-test. (I) Proportion of inner cell mass (ICM) cells in which SOX2 was detected among blastocysts. Statistical test: χ^2^. (J) Maximum projection of all sections of *z*-stack confocal imaging of SOX17 immunofluorescence and nuclear stain for a representative mock-infected and a representative ZIKV^UG^-infected blastocysts. (K) Average number of SOX17-positive cells in all embryos (sample sizes provided in the image panels) for each condition. Statistical test: unpaired *t*-test. (L) Proportion of ICM cells in which SOX17 was detected among blastocysts. Statistical test: χ^2^. ZIKV-E staining for these embryo stages can be found in Fig. 1. *n*, number of embryos. Scale bars: 50 µm.

Here, as in the published study (Tan et al., 2019), we examined whether CDX2-positive cells were also ZIKV-E positive. Next, we investigated whether the average number of embryonic cells per embryo is impacted by infection. We observed a significant (*P*<0.0001) reduction in the average number of total cells per embryo in ZIKV^UG^-infected blastocysts (*n*=15), compared with controls (*n*=9) (Fig. 2A). Additionally, we observed a significant decrease in average numbers of trophectoderm and ICM cells in infected blastocysts (both *P*<0.05) (Fig. 2B,C). These results strongly suggest that ZIKV infection disrupts cell fate specification in the blastocyst.

To investigate the ZIKV^UG^-induced phenotype further, we quantified the number of trophectoderm cells (as defined by outside position) expressing CDX2 in embryos. In ZIKV^UG^-exposed embryos (*n*=15), we observed a significant (*P*=0.002) decrease in the average number of outside cells expressing CDX2, compared with controls (*n*=9) (Fig. 2D,E). Moreover, the proportion of CDX2-positive outside cells was also significantly (*P*<0.001) lower in ZIKV^UG^-infected embryos (Fig. 2F). CDX2-negative trophectoderm cells could be considered to have morphological features of trophectoderm (i.e. outside position), but these cells had lost or failed to maintain expression of *Cdx2*. Given that *Cdx2* is essential for trophectoderm cell development (Strumpf et al., 2005), these data indicate that ZIKV infection interferes with trophectoderm fate.

We next investigated the effects of ZIKV^UG^ on ICM lineages. In unperturbed embryos, the epiblast marker SOX2 and primitive endoderm marker SOX17 are each detected in approximately half of all ICM cells (Plusa et al., 2008; Wicklow et al., 2014). In ZIKV^UG^-infected embryos (*n*=8), the average number of SOX2-positive cells was significantly (*P*=0.0003) reduced compared with controls (*n*=5) (Fig. 2G,H). However, the proportion of SOX2-positive ICM cells was not significantly impacted (*P*>0.05) (Fig. 2I), consistent with a smaller ICM (see Fig. 1C).

Finally, we evaluated the effect of ZIKV^UG^ on the expression of the primitive endoderm marker SOX17. Again, in ZIKV^UG^-infected embryos (*n*=7), the average number of SOX17-positive cells was significantly (*P*<0.05) reduced compared with controls (*n*=4) (Fig. 2J,K). Additionally, the proportion of SOX17-positive ICM cells was significantly (*P*<0.0001) reduced (Fig. 2L), suggesting that ZIKV^UG^ has a greater impact on the primitive endoderm than the epiblast under these conditions. As *Sox2* and *Sox17* are both essential for early development (Artus et al., 2011; Avilion et al., 2003), we conclude that ZIKV^UG^ infection is detrimental to the ICM lineages.

### ZIKV^UG^-induced lethality of mouse embryos at multiple preimplantation stages

**Fig. 3.**
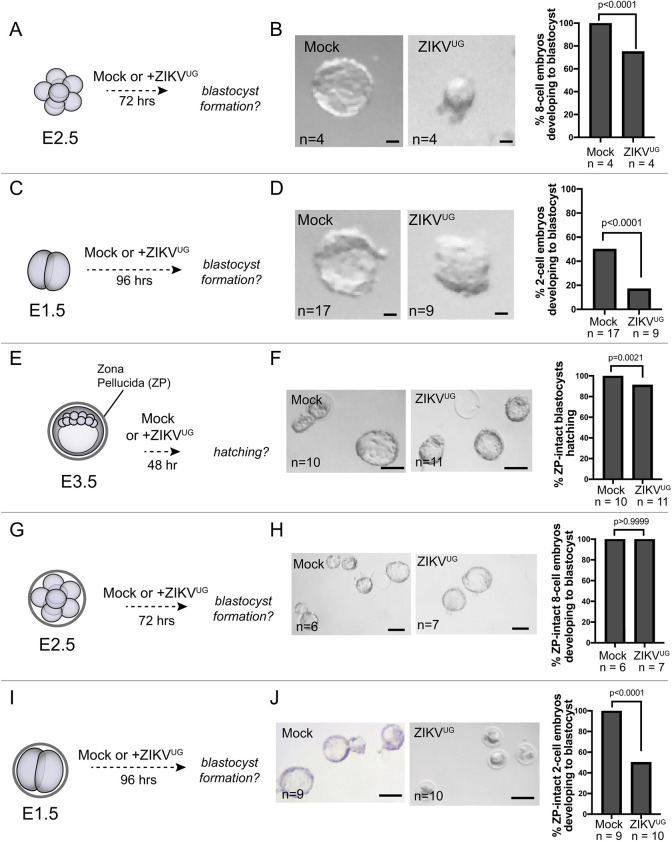
**ZIKV^UG^ infects embryos at multiple stages, and the ZP fails to protect embryos from ZIKV^UG^ at all stages.** (A) Schematic of the experimental design. Embryos were collected at the 8-cell stage (E2.5), the ZP was removed, and embryos were then cultured for 72 h in ZIKV^UG^-containing or mock medium. (B) Representative images of embryos cultured as described in A. Bar chart shows the proportion of embryos progressing to blastocyst stage. Statistical test: unpaired *t*-test. (C) Schematic of the experimental design. Embryos were collected at the 2-cell stage (E1.5), the ZP was removed, and embryos were then cultured for 96 h in ZIKV^UG^-containing or mock medium. (D) Representative images of embryos cultured as described in C. Bar chart shows the proportion of embryos progressing to blastocyst stage. Statistical test: unpaired *t*-test. (E,G,I) Schematic of the experimental design. Embryos were collected at the indicated stages, the ZP was left intact, and then embryos were cultured until the same developmental endpoint. (F) Representative images of embryos cultured as described in E. Bar chart shows the proportion of embryos progressing, evidenced by hatching from the ZP. Statistical test: unpaired *t*-test. (H) Representative images of embryos cultured as described in G. Bar chart shows the proportion of embryos progressing to blastocyst stage. Statistical test: unpaired *t*-test. (J) Representative images of embryos cultured as described in I. Bar chart shows the proportion of embryos progressing to blastocyst stage. Statistical test: unpaired *t*-test. *n*, number of embryos. Scale bars: 20 µm (B,D); 100 µm (F,H,J).

Up to this point, we had focused on the susceptibility of mouse blastocysts to ZIKV. However, the effects of ZIKV exposure on embryos during earlier development has not been investigated. We therefore evaluated the effects of ZIKV^UG^ on embryos in the absence of ZP at earlier stages. We first exposed ZP-free embryos to ZIKV^UG^ at the eight-cell stage (E2.5), and then cultured these to the same endpoint as for our prior studies (Fig. 3A). Following infection at the eight-cell stage, we observed a significant (*P*<0.0001) decrease in embryo viability after ZIKV^UG^ exposure (*n*=4), compared with controls (*n*=4) (Fig. 3B). Next, we examined the viability of ZIKV^UG^-infected embryos at the two-cell stage (E1.5) (Fig. 3C). At the two-cell stage, removal of the ZP decreased viability of controls, as anticipated (Nijs and Van Steirteghem, 1987). However, in ZIKV^UG^-exposed two-cell-stage embryos, viability was dramatically and significantly (*P*<0.0001) lower in ZIKV^UG^-exposed embryos (*n*=9) than in controls (*n*=17) (Fig. 3D). In the arrested ZIKV^UG^-exposed embryos, ZIKV-E could not be detected (Fig. S2), suggestive of a ZIKV-induced cytopathic effect at this stage. We conclude that ZP-free embryos are susceptible to ZIKV at several preimplantation stages.

### The zona pellucida fails to protect embryos from ZIKV-induced lethality

The results presented above and previously (Block et al., 2020; Tan et al., 2019) raise concern for the susceptibility of ZP-free preimplantation embryos to ZIKV infection. However, the embryo usually resides within the ZP until hatching at around E4.5 (McLaren, 1970). Several viruses have been observed to penetrate the ZP and infect preimplantation embryos (Gwatkin, 1963, 1967; Makarevich et al., 2007; Romeo et al., 2020), including viruses that are larger in diameter than ZIKV. Whether ZIKV^UG^ is capable of penetrating the ZP at multiple preimplantation stages has not been investigated.

To evaluate whether the ZP can protect preimplantation embryos from ZIKV^UG^-induced lethality, we exposed multiple stages of ZP-intact embryos to ZIKV^UG^ and then observed their *ex vivo* development. We noted a small, but significant (*P*=0.0021), decrease in the viability of ZP-intact blastocysts exposed to of ZIKV^UG^ (*n*=11) compared with controls (*n*=10) (Fig. 3E,F). However, the viability of ZP-intact eight-cell embryos was unaffected by ZIKV^UG^ exposure (*P*>0.05, *n*=6 mock infected and *n*=7 ZIKV^UG^ exposed) (Fig. 3G,H). Strikingly, the viability of ZP-intact two-cell embryos was severely compromised by ZIKV^UG^ exposure compared with controls, with around half of infected embryos arresting around the four-cell stage (*P*<0.0001, *n*=9 mock infected and *n*=10 ZIKV^UG^ exposed) (Fig. 3I,J). We conclude that the ZP is not a barrier to ZIKV^UG^-induced embryo lethality at the 2-cell stage.

### Two-cell embryos are vulnerable to multiple ZIKV strains

We and others (Tan et al., 2019) have observed that an African strain of ZIKV (ZIKV^UG^) can impact the development of preimplantation mouse embryos. A recent study showed that non-human primate preimplantation embryos are vulnerable to a ZIKV strain of Asian lineage (Block et al., 2020), but a comparative study of the two lineages has not been performed during preimplantation in any species. Both African and Asian strains of ZIKV can affect fetal and adult tissues (Anfasa et al., 2017; Gong et al., 2017; Shao et al., 2017; Simonin et al., 2016), but there is no clear consensus on which strain is the more virulent.

**Fig. 4.**
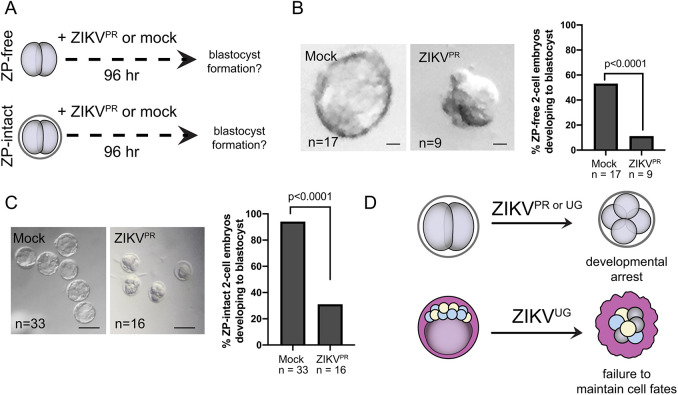
**Two-cell embryos are susceptible to the Asian lineage-derived ZIKV^PR^.** (A) Schematic of the experimental design. Embryos were harvested at the two-cell stage, and the ZP was either removed or left intact. Embryos were then exposed to ZIKV^PR^ for 96 h. (B) Representative images of ZP-free embryos cultured as described in A. Bar chart shows the proportion of embryos progressing to blastocyst stage. Statistical test: unpaired *t*-test. (C) Representative images of ZP-intact embryos cultured as described in A. Bar chart shows the proportion of embryos progressing to blastocyst stage. Statistical test: unpaired *t*-test. *n*, number of embryos. (D) Summary of key findings. Exposure of two-cell embryos to either strain of ZIKV leads to developmental arrest, even in the presence of the ZP. Exposure of ZP-free blastocysts to ZIKV^UG^ disrupts fetal and other cell fates, leading to embryo lethality. Scale bars: 20 µm (B); 100 µm (C).

We therefore evaluated whether the Asian-derived Puerto Rican strain of ZIKV (ZIKV^PR^) affects preimplantation. For this comparison, we focused on two-cell embryos with and without the ZP (Fig. 4A), because this appeared to be a uniquely susceptible stage. We noted that ZIKV^PR^ significantly (*P*<0.0001) disrupted development of ZP-free (Fig. 4B) and ZP-intact (Fig. 4C) embryos, and ZIKV-E could not be detected in the arrested embryos, again suggesting that ZIKV infection is cytopathic at this stage. These observations indicate that two-cell embryos are vulnerable to multiple ZIKV strains.

## DISCUSSION

According to the World Health Organization, ZIKV presents a major threat to human health, and its epidemic/pandemic potential is recognized (Eaton, 2021; Pierson and Diamond, 2020). Disturbingly, the *Aedes* mosquitoes that transmit ZIKV are among the top invasive species in the world (Cunze et al., 2018; Medlock et al., 2015). Moreover, ZIKV can also be transmitted sexually (Brooks et al., 2016; D'Ortenzio et al., 2016; Grischott et al., 2016), providing a second mechanism for global expansion. Indeed, the capacity of human-borne pathogens to spread beyond control are unfortunately all too familiar. ZIKV vaccines are under development, but are not yet widely available (Pattnaik et al., 2020).

We have shown that, in the absence of the ZP, all three blastocyst lineages are susceptible to infection, including the fetal lineage. Importantly, increased apoptosis was detected in ZIKV-infected mouse blastocysts lacking the ZP (Tan et al., 2019), providing a possible mechanism for the observed disruptions to the blastocyst lineages (Fig. 4D). However, in future studies, apoptosis should be examined alongside lineage markers in embryos exposed to ZIKV to determine whether ZIKV impacts apoptosis in all blastocyst lineages in the absence of the ZP, and whether ZIKV impacts apoptosis or blastocyst lineage composition in the presence of the ZP. Alternatively, embryo lethality and defects in lineage specification could be the indirect effect of an as-yet-unidentified component of Vero cell-conditioned medium that is produced in response to ZIKV infection, as purified virus was not used in this or previous studies (Block et al., 2020; Tan et al., 2019). Nevertheless, we and other groups detect the ZIKV-E protein within blastomeres, suggesting that virus is entering and replicating within preimplantation embryos. Future studies in human embryos should make use of purified virus to address this caveat.

As others have shown (Block et al., 2020; Tan et al., 2019), we also observed ZIKV-induced blastocyst lethality in the absence of the ZP. This is important because embryos typically hatch from the ZP at the late blastocyst stage, and therefore human embryos could be vulnerable to ZIKV infection at this stage. Although the ZP could protect embryos at some stages prior to the blastocyst stage, we have observed that two-cell embryos with intact ZPs are still vulnerable to ZIKV. We do not yet understand why the two-cell stage is particularly vulnerable to infection, but we consider several possibilities, including ultrastructural features of the ZP, heightened expression of novel ZIKV entry proteins, and/or vulnerability of the maternal-to-zygotic transition in gene expression. We also do not know how long ZIKV remains viable and infectious in the human reproductive system or in culture. Nor do we know whether and when ZIKV can breach the ZP of human embryos, and these are all potential topics of future investigation. Overall, our observations reinforce concerns that human-borne ZIKV infection could threaten viability of human early embryos, impacting human miscarriage rates and fertility (Duggal et al., 2018; Moreira et al., 2016), meriting further study.

We have also demonstrated for the first time that ZIKV can infect the primitive endoderm lineage in the absence of the ZP. In mouse, the primitive endoderm-derived extra-embryonic endoderm plays essential roles in the development of the brain, intestine, heart, germ cells and blood (Belaoussoff et al., 1998; de Sousa Lopes et al., 2004; Kwon et al., 2008; Madabhushi and Lacy, 2011; Mao et al., 2010; Thomas and Beddington, 1996). In human embryos, the epiblast and primitive endoderm (known as hypoblast) are physically adjacent, as in mouse (Ralston, 2018), suggesting conservation of function. However, whether human hypoblast damage could contribute to birth defects has not been investigated.

Our observations provide rationale for examining the susceptibility of human epiblast and primitive endoderm to ZIKV infection. As studies of human preimplantation embryos are, necessarily, performed *ex vivo*, our findings and conditions are directly applicable to this experimental setting. An additional benefit of *ex vivo* embryo study is that it permits evaluation of the effects of viral infection on embryos without the influence of the maternal immune system. This is important because the maternal immune response to ZIKV can vary among species, mouse strains and individuals (Ander et al., 2019; Khan et al., 2016). Our observations establish the intrinsic susceptibility of preimplantation embryos to infection by both Asian and African ZIKV lineages, and warrant follow-up studies to determine whether and how sexually transmitted ZIKV impacts human fertility and pregnancy outcomes.

## MATERIALS AND METHODS

### Animal use

All animal research was conducted in accordance with the guidelines and approval of the Michigan State University Institutional Animal Care and Use Committee. Experiments were performed using male and female CD-1 mice, at least 6-8 weeks of age. Animals were maintained on a 12-h light/dark cycle with *ad libitum* access to food and water.

### Virus propagation and preparations

Vero cells (ATCC, CRL-1586) were cultured in 75-cm^2^ filtered cap flasks to 90-95% confluency in 10% fetal bovine serum (FBS) (Hyclone, SH30396)-EMEM (ATCC, 30-2003) in 5% CO_2_ at 37°C. Cells were washed with 5-10 ml of Dulbecco's phosphate-buffered saline (Life Technologies, 14040133). Before infection, one flask was used to determine cell count. The remaining flasks of Vero cells were infected for 1 h at a multiplicity of infection (MOI) of 0.01 in 5 ml of 2% FBS-EMEM at 5% CO_2_ at 37°C, rocking every 15 min, after which 4 ml 10% FBS-EMEM was added. Cell culture supernatants were collected 40-48 h later, and then centrifuged for 10 min 1300 ***g*** at 4°C. Supernatants were pooled, and 1-ml aliquots were then stored at −80°C. For negative control experiments, Vero cell medium or Vero cell-conditioned medium was used, as has been done previously (Block et al., 2020; Scaturro et al., 2018; Wells et al., 2016).

### Titration by plaque assay

Vero cells (ATCC, CRL-1586) were cultured in a 6-well plate to 100% confluency in 10% FBS-EMEM in 5% CO_2_ at 37°C. Cells were washed in EMEM without FBS and infected with ZIKV^PR^ (PRVABC, ATCC, VR-1843) and ZIKV^UG^ (MR776, ATCC, VR-1838) diluted to 10^2^-10^6^ in 500 ml 2% FBS-EMEM. Cells were infected or mock-infected for 15 min, with rocking at room temperature, and then incubated for 45 min in 5% CO_2_ at 37°C. Cells were subsequently overlaid with 4 ml 2% methylcellulose (Sigma-Aldrich, M0512) in EMEM, and then incubated for 6-7 days. The overlay was then removed, and cells were then fixed in 4% paraformaldehyde (Electron Microscopy Sciences,15710). Vero cells were stained with 0.1% Crystal Violet (Sigma-Aldrich, C0775) solution prepared in 20% ethanol and 4% paraformaldehyde. Crystal Violet stain was washed gently with water until the water was clear. Plaques were allowed to dry for 1-24 h and counted under a transilluminator to determine viral titer (pfu/ml).

### Embryo ZIKV infection

Before embryo culture, KSOM medium (Millipore, MR-121-D) and EmbryoMax Filtered Light Mineral Oil (Millipore, ES-005-C) were equilibrated overnight in 5% CO_2_ at 37°C. CD-1 embryos were collected with M2 medium via the oviduct at E1.5 and E2.5 and via the uterine horn at E3.5 days post-copulatory plug. The ZP remained intact or was removed two embryos at a time in two or three drops of 60 µl Acidic Tyrode's Solution (Millipore, MR-004-D), and embryos were then washed twice in M2 medium. Embryos were then cultured for 24, 48 or 96 h in 5% CO_2_ at 37°C in 20 µl KSOM with ZIKV^UG^, ZIKV^PR^ or equivalent concentration mock medium to create the inoculum. Final concentrations of ZIKV^UG^ and ZIKV^PR^ were 6×10^4^ pfu/ml. Mock medium was either Vero cell medium or Vero cell-conditioned medium. Embryo viability was equivalent among embryos cultured in either mock medium.

### Immunofluorescence and confocal microscopy

Embryos were fixed in 4% formaldehyde for 10 min at room temperature, permeabilized with 0.5% Triton X-100 (Sigma-Aldrich, X100) in PBS for 30 min at room temperature, and blocked with 10% FBS and 0.1% Triton X-100 in PBS for 1 h at room temperature or longer at 4°C. Primary antibodies were prepared in blocking buffer, and embryos and cells were incubated at 4°C overnight with various combinations of the following primary antibodies: goat anti-mouse anti-mCDX2 (1:200, BioGenex, CDX2-88), goat anti-SOX2 (1:2000, Neuromics, GT15098), goat anti-hSOX17 (1:2000, R&D Systems, AF1924) or rabbit anti-ZIKV-E (1:500, GeneTex, GTX133314). Embryos and cells were then washed with blocking buffer for 30 min, incubated with the following secondary antibodies for 1 h: donkey anti-goat Alexa 488 (1:400, Invitrogen, A-11055), donkey anti-rabbit Cy3 (1:400, Jackson ImmunoResearch, 711-165-152), donkey anti-mouse Cy3 (1:400, Jackson ImmunoResearch, 715-165-150) and donkey anti-rabbit Alexa 647 (1:400, Jackson ImmunoResearch, 711-606-152), and washed with blocking buffer for 30 min. Nuclei were stained with DRAQ5 (Cell Signaling Technology, 4084) or Hoechst 33342 (Thermo Fisher Scientific, 62249). Embryo and cell images were captured on a Nikon a1 confocal laser scanning microscope using 60× Plan Apo IR water objective (numerical aperture 1.27 WI). Every embryo was imaged by collecting a complete *z*-stack, with 5 µm between each image.

### Image analysis for embryos

Images were analyzed with Fiji ImageJ software. Cells were counted manually in each plane of each *z*-stack, and the resulting data were imported into Excel or GraphPad software. Graphs were generated with GraphPad. Statistical significance was evaluated using χ^2^ tests or unpaired *t*-tests, as indicated in figure legends.

## Supplementary Material

Supplementary information

Reviewer comments
