# Supplementary material for: The fetal lineage is susceptible to Zika virus infection within days of fertilization
Source: Development. 2022 Jul 28;149(14):dev200501. doi: 10.1242/dev.200501 (PMC9382896; doi:10.1242/dev.200501)
Supplement: Supplementary information [file develop-149-200501-s1.pdf]

Watts and Ralston, Fig. S1 for Fig 1.

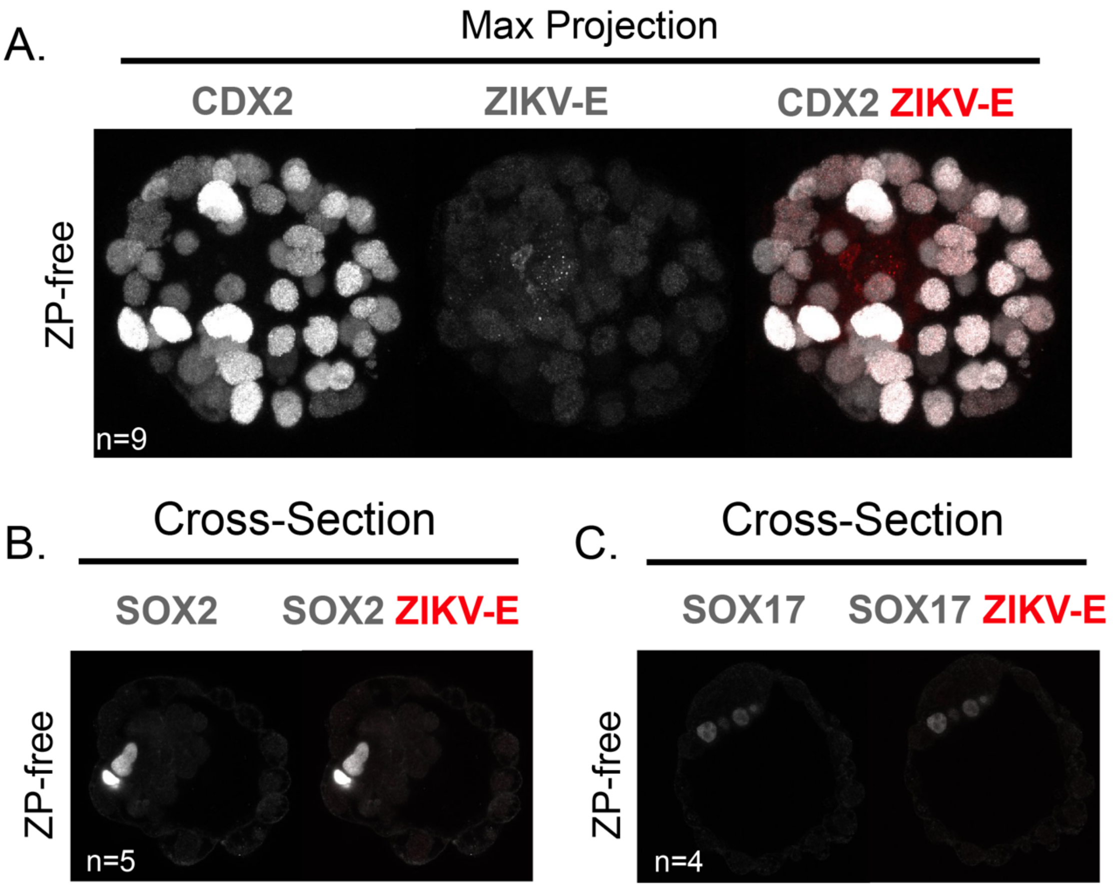

Fig. S1. Negative control for ZIKV-E immunofluorescence experiments.

A) Embryos were not exposed to ZIKV, and were then immunofluorescently stained with antibodies to detect CDX2 and anti-ZIKV-E, demonstrating ZIKV-dependent ZIKV-E signal detected in Fig. 1.

B) Similar to panel A, uninfected embryos were immunofluorescently stained with antibodies to detect SOX2 and anti-ZIKV-E.

C) Uninfected embryos were immunofluorescently stained with antibodies to detect SOX17 and anti-ZIKV-E.

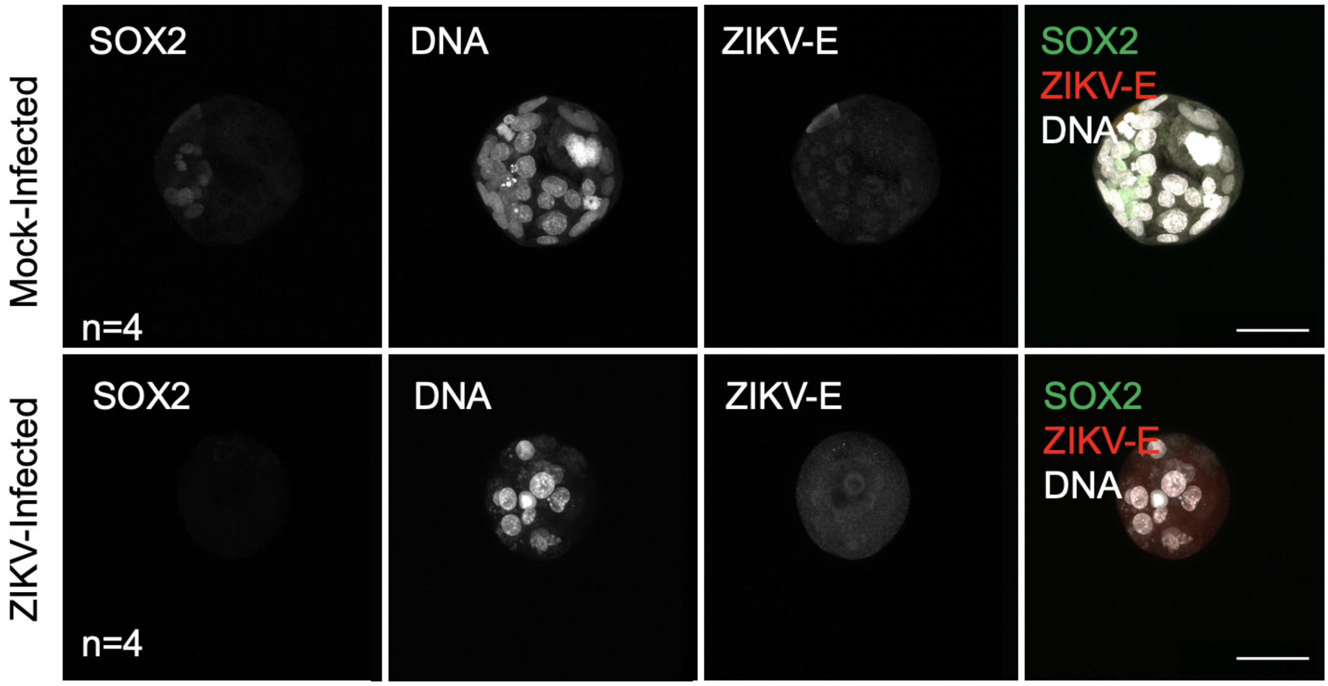

Fig. S2. ZIKV-E is not detected within ZIKV-exposed embryos when infection begins at the two-cell stage. Control embryos are shown in the upper panels and ZIKV-exposed embryos, which have failed to develop, are shown in the lower panels. Sample size is indicated and scale bar: 50 µm.
