# Supplementary material for: The fetal lineage is susceptible to Zika virus infection within days of fertilization
Source: Development. 2022 Jul 28;149(14):dev200501. doi: 10.1242/dev.200501 (PMC9382896; doi:10.1242/dev.200501)
Supplement: Reviewer comments [file develop-149-200501_review_history.pdf]

The fetal lineage is susceptible to Zika virus infection within days of fertilization

Jennifer L. Watts and Amy Ralston
DOI: 10.1242/dev.200501

Editor: Liz Robertson

Review timeline
Original submission: 7 January 2022
Editorial decision: 25 February 2022
First revision received: 24 May 2022
Accepted: 9 June 2022

Original submission

First decision letter

MS ID#: DEVELOP/2022/200501

MS TITLE: The fetal lineage is susceptible to Zika virus infection within days of fertilization

AUTHORS: Jennifer L Watts and Amy Ralston

I have now received all the referees reports on the above manuscript, and have reached a decision. The referees' comments are appended below, or you can access them online: please go to BenchPress and click on the 'Manuscripts with Decisions' queue in the Author Area.

The overall evaluation is positive and we would like to publish a revised manuscript in Development, provided that the referees' comments can be satisfactorily addressed. Please attend to all of the reviewers' comments in your revised manuscript and detail them in your point-by-point response. If you do not agree with any of their criticisms or suggestions explain clearly why this is so.

We are aware that you may currently be unable to access the lab to undertake experimental revisions. If it would be helpful, we encourage you to contact us to discuss your revision in greater detail. Please send us a point-by-point response indicating where you are able to address concerns raised (either experimentally or by changes to the text) and where you will not be able to do so within the normal timeframe of a revision. We will then provide further guidance. Please also note that we are happy to extend revision timeframes as necessary.

Reviewer 1

Advance summary and potential significance to field

In their manuscript, “The fetal lineage is susceptible to Zika virus infection within days of fertilization“
Watts and Ralston investigate Zika virus infection of the preimplantation mouse embryo. This is an important question as there are scant data regarding mammalian embryo infection in the maternal reproductive tract despite our knowing that Zika is sexually transmitted. Through careful quantification of specific lineages in the blastocyst, the authors demonstrate that Zika infects all

© 2022. Published by The Company of Biologists under the terms of the Creative Commons Attribution License (https://creativecommons.org/licenses/by/4.0/).

lineages of the blastocyst including the cells that will become the embryo proper. The strengths of the work include its examination of specific cell lineages using endogenous markers of cell fate, its use of distinct Zika strains, and its rigor including the use of proper controls and careful quantification. The work advances the field by pinpointing the cell types and time points where Zika acts. This is critical as it reveals when and where the field must focus in human embryos to understand the etiology of Zika's impact. Furthermore, the work is of interest to readers of Development as it approaches a public health threat from a classic developmental standpoint (fundamental cell fate specification). While the weaknesses are minor, there is an editorial question regarding whether a more mechanistic understanding is necessary at this time.

Comments for the author

1) Is there increased apoptosis in all lineages that are Zika infected? The authors suggest there is in the discussion, where they cite the Tan work. But that work did not examine all lineages within the blastocyst so it is unclear whether Zika interferes with cell fate in the distinct lineages through a potentially common mechanism or clearly distinct ones.
2) In Figure 2, the supplemental figure showing the decreased proportion of CDX2, SOX2 and SOX17 cells in the Zika infected embryos provides more compelling evidence that Zika interferes with fate than the numbers of such cells reported in Figure 2. Thus, it seems including the S2 graphs in Fig 2 D, E, and F would be warranted. If there are space constraints, then the current Fig 2 D, E and F graphs could become S2.
3) In Figure 3 E-H, the layout would be far more intuitive to follow if it paralleled A-D with the schematic horizontally aligned with the relevant data.
4) In Fig 4D, the bottom panel should specify ZIKVUG (not ZIKV generally) leads to failure to maintain cell fates whereas the top panel can show ZIKV (as both UG and PR were examined). The authors did not examine the specific blastocyst lineages with Zika-PR.

Reviewer 2

Advance summary and potential significance to field

The authors present an interesting finding that in mouse embryos ZIKV can infect all three lineages of preimplantation stage embryos and that embryos prior to the blastocyst stage undergo arrest following infection. This is an interesting observation and a progression from previous findings. In general the data could be better presented to strengthen their interesting conclusions and revisions to the text would be helpful to place in context the work and caveats of the findings that could be followed up in subsequent studies. This would be very helpful to make it clear what are the main conclusions that can be drawn and the implications for humans and which of the observations made are more speculative and open to other interpretations given the limitations of the study.

Comments for the author

It would be informative if the authors could speak to the use of the Mock control in the manuscript and expand a little on this in the results section and the use of other controls in the discussion that would provide even more confidence in drawing conclusions from the results presented.. In the methods section they state that no developmental differences were observed in the mock treated embryos, but how was this evaluated and compared to untreated controls? What is the nature of the mock medium? Had they considered other types of controls (i.e. viral transduction with another type of virus such as lentivirus that may have been concentrated in a similar way (i.e. similar titter and basal media)? Or inactivated ZIKV? The use of alternative controls would be helpful to discuss in the discussion section and whether there may be any limitations in the conclusions drawn and subsequent studies that could be proposed for the future.
Embryos in utero would have an intact zona pellucida therefore the conclusions drawn from Figure 3 are more likely to be relevant to in vivo conditions. I therefore wondered if the authors had evaluated the total cell number and lineage markers such as CDX2, SOX2 or SOX17 in these embryos? If so it would be helpful to include this data. Do they have evidence of the infection of these embryos by ZIKV similar to figure 1C-E that could also be included? If so this would further support their conclusions that the zona pelucida is not a barrier to infection.

© 2022. Published by The Company of Biologists under the terms of the Creative Commons Attribution License (https://creativecommons.org/licenses/by/4.0/).

I think the discussion section needs to be revised to include a paragraph on the some of the major caveats of their study. It is understandable to have caveats given the challenges of the experiments but these needs to be presented up front. For example the conclusions drawn from the embryos where the ZP has been removed need to be put into the context that ZP removal does not occur in vivo. It is also unclear if there was ZIKV infection in the embryos with intact ZP from the data presented. If embryos arrest at the early cleavage stages following sexual transmission of ZIKV as the authors are suggesting, then the embryos should never go on to have abnormalities in any blastocyst lineages because they would never reach the blastocyst stage. The authors should revise the paragraph starting on lines 254-261 accordingly or make clearer what they would expect in humans in the discussion section.
When writing about the statistical significance throughout the manuscript it would be helpful if the authors could include the significance numbers and n numbers in the main body of the results section and to expand on these findings.
In figures 3 and 4 do the authors have images confirming ZIKV infection similar to figure 1C-E that could also be included?
In Figure 1C-E it would be helpful to have the DAPI nuclear panels included. This is because the embryos shown are clearly affected negatively as a consequence of ZIKV exposure and it is a little challenging to orient the boundary between cells. It would be ideal if the infected embryos were stained with a cell membrane marker but I appreciate that these are challenging experiments to set up and it would be preferable to include a marker that the authors may have already evaluated and may have to hand.
For some of the panels of figures the images a blurry (e.g. Figure 1B, 3B, 3D, 4B) and I wondered if the authors may have images that were taken where embryos were more in focus and of higher resolution that could be included instead? This would be helpful to see at what stage the embryos may have arrested.
Is it known how long ZIKV remains infections in the conditions used in the study? What are the implications of this for some of the discussion points?

Minor:
Line 100: Correct to: During preimplantation development…
Line 102: Correct to: …protects embryos from viral infection…
Lines 109 -110: Clarify what these studies found, in what way was ZIKV found to be harmful?
Lines 113-115: Amend this to focus on the positives of the study, for example: Here we present a systematic evaluation of lineage-associated molecular markers of early preimplantation mouse embryos exposed to ZIKV and find that ….
Lines 140-141: state clearly the role of the primitive endoderm at the moment it is written in a vague way.
Line 171: Change the word “normal”.
Line 177: change to: …with a failure to maintain cell fates.
Line 415: I think should be Acidic Tyrode’s solution and the catalogue number here, and throughout the methods section for reagents, needs to be included.
In general, I would suggest to the authors to highlight more positively the strengths of their work without being overly negative about previous studies. For example, on line 150 this section could be started in a different way to highlight the findings of the current study.

First revision

<u>Author response to reviewers' comments</u>

Reviewer 1 Advance Summary and Potential Significance to Field:
In their manuscript, “The fetal lineage is susceptible to Zika virus infection within days of fertilization“ Watts and Ralston investigate Zika virus infection of the preimplantation mouse embryo. This is an important question as there are scant data regarding mammalian embryo infection in the maternal reproductive tract despite our knowing that Zika is sexually transmitted. Through careful quantification of specific lineages in the blastocyst, the authors demonstrate that Zika infects all lineages of the blastocyst, including the cells that will become the embryo proper. The strengths of the work include its examination of specific cell

© 2022. Published by The Company of Biologists under the terms of the Creative Commons Attribution License (https://creativecommons.org/licenses/by/4.0/).

lineages using endogenous markers of cell fate, its use of distinct Zika strains, and its rigor including the use of proper controls and careful quantification. The work advances the field by pinpointing the cell types and time points where Zika acts. This is critical as it reveals when and where the field must focus in human embryos to understand the etiology of Zika's impact. Furthermore, the work is of interest to readers of Development as it approaches a public health threat from a classic developmental standpoint (fundamental cell fate specification). While the weaknesses are minor, there is an editorial question regarding whether a more mechanistic understanding is necessary at this time.

We thank Reviewer 1 for a very thoughtful reading of our manuscript.

Reviewer 1 Comments for the Author:

1) Is there increased apoptosis in all lineages that are Zika infected? The authors suggest there is in the discussion, where they cite the Tan work. But that work did not examine all lineages within the blastocyst so it is unclear whether Zika interferes with cell fate in the distinct lineages through a potentially common mechanism or clearly distinct ones.

We did not have the chance to perform this analysis before the first author departed the lab for her postdoctoral fellowship. The transition was further complicated by the pandemic. However, we address the reviewer's point, which is a good one, on lines 270-273 of the revised manuscript.

2) In Figure 2, the supplemental figure showing the decreased proportion of CDX2, SOX2 and SOX17 cells in the Zika infected embryos provides more compelling evidence that Zika interferes with fate than the numbers of such cells reported in Figure 2. Thus, it seems including the S2 graphs in Fig 2 D, E, and F would be warranted. If there are space constraints, then the current Fig 2 D, E and F graphs could become S2.

This is a good suggestion, and we have moved the supplemental graphs to Figure 2 in the revised manuscript.

3) In Figure 3 E-H, the layout would be far more intuitive to follow if it paralleled A-D with the schematic horizontally aligned with the relevant data.

We have made the requested change.

4) In Fig 4D, the bottom panel should specify ZIKVUG (not ZIKV generally) leads to failure to maintain cell fates whereas the top panel can show ZIKV (as both UG and PR were examined). The authors did not examine the specific blastocyst lineages with Zika-PR.

Done.

Reviewer 2 Advance Summary and Potential Significance to Field:
The authors present an interesting finding that in mouse embryos ZIKV can infect all three lineages of preimplantation stage embryos and that embryos prior to the blastocyst stage undergo arrest following infection. This is an interesting observation and a progression from previous findings. In general the data could be better presented to strengthen their interesting conclusions and revisions to the text would be helpful to place in context the work and caveats of the findings that could be followed up in subsequent studies. This would be very helpful to make it clear what are the main conclusions that can be drawn and the implications for humans and which of the observations made are more speculative and open to other interpretations given the limitations of the study.

We thank Reviewer 2 for the very detailed reading and helpful suggestions.

Reviewer 2 Comments for the Author:
It would be informative if the authors could speak to the use of the Mock control in the manuscript and expand a little on this in the results section and the use of other controls in the discussion that would provide even more confidence in drawing conclusions from the results presented.. In the methods section they state that no developmental differences were

© 2022. Published by The Company of Biologists under the terms of the Creative Commons Attribution License (https://creativecommons.org/licenses/by/4.0/).

observed in the mock treated embryos, but how was this evaluated and compared to untreated controls? What is the nature of the mock medium? Had they considered other types of controls (i.e. viral transduction with another type of virus such as lentivirus that may have been concentrated in a similar way (i.e. similar titter and basal media)? Or inactivated ZIKV? The use of alternative controls would be helpful to discuss in the discussion section and whether there may be any limitations in the conclusions drawn and subsequent studies that could be proposed for the future.

This is an interesting point that we discussed a lot during the study. We have added additional information about our controls, the reasoning behind these controls, and caveats of controls to the manuscript Results and Discussion sections, lines: 126-130 and 273-280.

Embryos in utero would have an intact zona pellucida therefore the conclusions drawn from Figure 3 are more likely to be relevant to in vivo conditions. I therefore wondered if the authors had evaluated the total cell number and lineage markers such as CDX2, SOX2 or SOX17 in these embryos? If so it would be helpful to include this data. Do they have evidence of the infection of these embryos by ZIKV similar to figure 1C-E that could also be included? If so this would further support their conclusions that the zona pelucida is not a barrier to infection.

We were unable to perform these experiments due to the pandemic disruption. However, we now touch on this issue in the revised manuscript, lines 272-273.

I think the discussion section needs to be revised to include a paragraph on the some of the major caveats of their study. It is understandable to have caveats given the challenges of the experiments but these needs to be presented up front. For example the conclusions drawn from the embryos where the ZP has been removed need to be put into the context that ZP removal does not occur in vivo. It is also unclear if there was ZIKV infection in the embryos with intact ZP from the data presented. If embryos arrest at the early cleavage stages following sexual transmission of ZIKV as the authors are suggesting, then the embryos should never go on to have abnormalities in any blastocyst lineages because they would never reach the blastocyst stage. The authors should revise the paragraph starting on lines 254-261 accordingly or make clearer what they would expect in humans in the discussion section.

We have added this discussion on lines 283-293 of the revised manuscript.

When writing about the statistical significance throughout the manuscript it would be helpful if the authors could include the significance numbers and n numbers in the main body of the results section and to expand on these findings.

We have made the requested changes throughout the manuscript.

In figures 3 and 4 do the authors have images confirming ZIKV infection similar to figure 1C-E that could also be included?

We were unable to detect ZIKV using the anti-ZIKV-E antibody in these experiments. We presume that this is a cytopathic effect of ZIKV, wherein rapid cell lethality precludes the replication of ZIKV to the point where it could be detected by immunofluorescence. We explain this in the revised manuscript on lines 211 and 252.

In Figure 1C-E it would be helpful to have the DAPI nuclear panels included. This is because the embryos shown are clearly affected negatively as a consequence of ZIKV exposure and it is a little challenging to orient the boundary between cells. It would be ideal if the infected embryos were stained with a cell membrane marker but I appreciate that these are challenging experiments to set up and it would be preferable to include a marker that the authors may have already evaluated and may have to hand.

We have included the requested figures.

© 2022. Published by The Company of Biologists under the terms of the Creative Commons Attribution License (https://creativecommons.org/licenses/by/4.0/).

For some of the panels of figures the images a blurry (e.g. Figure 1B, 3B, 3D, 4B) and I wondered if the authors may have images that were taken where embryos were more in focus and of higher resolution that could be included instead? This would be helpful to see at what stage the embryos may have arrested.

Unfortunately, these are the best images we were able to collect. However, we have made every effort to adjust the resolution of the final images for publication.

Is it known how long ZIKV remains infections in the conditions used in the study? What are the implications of this for some of the discussion points?

This is unknown. We now discuss this on lines 291-292 of the revised manuscript.

Minor:

Line 100: Correct to: During preimplantation development…
Done. Now lines 100-101.

Line 102: Correct to: …protects embryos from viral infection…
Done. Now line 102.

Lines 109 -110: Clarify what these studies found, in what way was ZIKV found to be harmful?
Clarified embryo lethality on lines 109-110.

Lines 113-115: Amend this to focus on the positives of the study, for example: Here we present a systematic evaluation of lineage-associated molecular markers of early preimplantation mouse embryos exposed to ZIKV and find that ….
Done. Now lines 114-116.

Lines 140-141: state clearly the role of the primitive endoderm at the moment it is written in a vague way.
Done. Now lines 146-150.

Line 171: Change the word "normal".
Changed to "unperturbed." Now on line 181.

Line 177: change to: …with a failure to maintain cell fates.
This section was revised in the process of incorporating discussion of significance as requested above. Now lines 189-196

Line 415: I think should be Acidic Tyrode's solution and the catalogue number here, and throughout the methods section for reagents, needs to be included.
Done. Changes made throughout the Methods section.

In general, I would suggest to the authors to highlight more positively the strengths of their work without being overly negative about previous studies. For example, on line 150 this section could be started in a different way to highlight the findings of the current study.
Great point! We have attempted to remove any overly negative language. Again, we might blame the pandemic-induced human isolation… Nevertheless, this specific example can be found on lines 161-163 of the revised manuscript.

© 2022. Published by The Company of Biologists under the terms of the Creative Commons Attribution License (https://creativecommons.org/licenses/by/4.0/).

Second decision letter

MS ID#: DEVELOP/2022/200501

MS TITLE: The fetal lineage is susceptible to Zika virus infection within days of fertilization

AUTHORS: Jennifer L Watts and Amy Ralston
ARTICLE TYPE: Research Report

I am happy to tell you that your manuscript has been accepted for publication in Development, pending our standard ethics checks.

Reviewer 1

Advance summary and potential significance to field

The authors have satisfactorily addressed my concerns.

Comments for the author

no further suggestions

Reviewer 2

Advance summary and potential significance to field

The manuscript is much improved and the authors have addressed all of my points.

Comments for the author

I have no further suggestions.

© 2022. Published by The Company of Biologists under the terms of the Creative Commons Attribution License (https://creativecommons.org/licenses/by/4.0/).
